# Donor-derived CD19 CAR-T cell therapy of relapse of CD19-positive B-ALL post allotransplant

**DOI:** 10.1038/s41375-020-01056-6

**Published:** 2020-10-19

**Authors:** Cheng Zhang, Xiao-Qi Wang, Rong-Li Zhang, Fang Liu, Yi Wang, Zhi-Ling Yan, Yong-Ping Song, Ting Yang, Ping Li, Zhen Wang, Ying-Ying Ma, Lei Gao, Yao Liu, Li Gao, Pei-Yan Kong, Jun Liu, Xu Tan, Jiang F. Zhong, Yu-Qing Chen, Ai-Bin Liang, Jin-Hua Ren, Zhen-Yu Li, Jiang Cao, Quan-Li Gao, Jian Zhou, Ying Gao, Ding Zhang, Fang-Yi Fan, Ming-Zhe Han, Robert Peter Gale, Xi Zhang

**Affiliations:** 1 Medical Center of Hematology, Xinqiao Hospital, State Key Laboratory of Trauma, Burn and Combined Injury, Army Medical University, Chongqing, 400037 China; 2 State Key Laboratory of Experimental Hematology, Institute of Hematology & Blood Disease Hospital, Chinese Academy of Medical Sciences & Peking Union Medical College, Tianjin, 300020 China; 3 The General Hospital of Western Theater Command, Chengdu, 610083 China; 4 Department of Hematology, the Shaanxi Provincial People’s Hospital, Xi’an, 710068 China; 5 Department of Hematology, the Affiliated Hospital of Xuzhou Medical University, Xuzhou, 221002 Jiangsu China; 6 Department of Hematology, Henan Cancer Hospital, Zhengzhou, 450008 Henan China; 7 Department of Hematology, Fujian Institute of Hematology, Fujian Provincial Key Laboratory of Hematology, Fujian Medical University Union Hospital, Fuzhou, Fujian, 350001 China; 8 Department of Hematology, Tongji Hospital of Tongji University, Shanghai, 200065 China; 9 Department of Hematology, Henan Provincial People’s Hospital, People’s Hospital of Henan University, Zhengzhou, 450003 Henan China; 10 Department of Otolaryngology, Keck School of Medicine, University of Southern California, Los Angeles, CA USA; 11 Haematology Research Centre, Department of Immunology and Inflammation, Imperial College London, London, UK

**Keywords:** Cancer immunotherapy, Acute lymphocytic leukaemia

## Abstract

Safety and efficacy of allogeneic anti-CD19 chimeric antigen receptor T cells (CAR-T cells) in persons with CD19-positive B-cell acute lymphoblastic leukemia (B-ALL) relapsing after an allotransplant remain unclear. Forty-three subjects with B-ALL relapsing post allotransplant received CAR-T cells were analyzed. 34 (79%; 95% confidence interval [CI]: 66, 92%) achieved complete histological remission (CR). Cytokine release syndrome (CRS) occurred in 38 (88%; 78, 98%) and was ≥grade-3 in 7. Two subjects died from multiorgan failure and CRS. Nine subjects (21%; 8, 34%) developed ≤grade-2 immune effector cell-associated neurotoxicity syndrome (ICANS). Two subjects developed ≤grade-2 acute graft-*versus*-host disease (G*v*HD). 1-year event-free survival (EFS) and survival was 43% (25, 62%). In 32 subjects with a complete histological remission without a second transplant, 1-year cumulative incidence of relapse was 41% (25, 62%) and 1-year EFS and survival, 59% (37, 81%). Therapy of B-ALL subjects relapsing post transplant with donor-derived CAR-T cells is safe and effective but associated with a high rate of CRS. Outcomes seem comparable to those achieved with alternative therapies but data from a randomized trial are lacking.

## Introduction

Persons experiencing B-cell acute lymphoblastic leukemia (B-ALL) relapse after an allogeneic hematopoietic cell transplant are typically treated by stopping immune suppression, receiving a donor lymphocyte infusion (DLI) and/or receiving a second transplant from the same or a different donor. The outcomes of these interventions are unsatisfactory [1–3].

Autologous anti-CD19 chimeric antigen receptor T cells (CAR-T cells) are an effective therapy for advanced CD19-positive B-ALL, often followed by an allotransplant [4–8]. Cumulative incidence of relapse (CIR) and event-free survival (EFS) of persons receiving CAR-T cells without an allotransplant are typically short [9]. Allogeneic anti-CD19 CAR-T cells receive activation signals from T-cell receptors (TCRs) to target cell alloantigens and from CD19 on leukemia cells. This dual signaling may increase the anti-leukemia efficacy compared with autologous CAR-T cells. Allogeneic anti-CD19 CAR-T cells can be developed from donor T cells in allotransplant recipients who relapse. However, the safety and efficacy of this approach are unknown.

We determined the safety and efficacy of donor-derived anti-CD19 CAR-T cells in 43 subjects with CD19-positive B-ALL relapsing after an allotransplant. Outcomes from data reported in this setting with DLI and with a second allotransplant were then compared. Results of donor-derived anti-CD19 CAR-T cells seem at least comparable if not better than these alternatives. However, these results can be tested only in a randomized trial.

## Methods

### Subjects and data collection

Forty-three subjects with CD19-positive B-ALL who received an allotransplant, had a bone marrow relapse and received donor-derived anti-CD19 CAR-T cells from July 2015 to March 2019 were enrolled. Major inclusion criteria included (1) Eastern Cooperative Oncology Group performance score ≤grade-2; (2) estimated survival >3 months; (3) no prior acute graft-*versus*-host disease; and (4) refusal to receive a second allotransplant. Posttransplant relapse was defined as >5% bone marrow blasts or a positive measurable residual disease (MRD) test after ≥2 prior posttransplant MRD tests. Details of MRD testing are reported [6, 8]. Data were extracted from the electronic medical records of subjects enrolled in ChiCTR-OOC-16008447, protocol number: ChIECRCT-20160022 and ChiCTR-OIC-17012374, protocol number: XYFY2017-KL033-01. The study was approved by the Ethics Committee of the Army Medical University.

### CD19-targeting CAR-T manufacturing

**Fig. 1:**
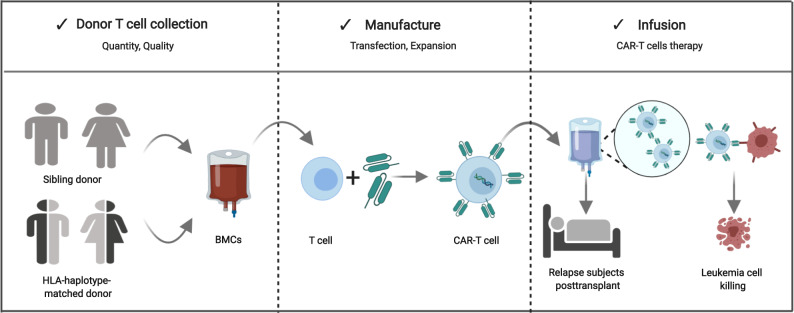
The procedure of donor-derived CD19 CAR-T cells. The BMCs were apheresised from sibling donor or HLA-haplotype-matched donor, then the T cells were selected and transfected with lentivirus to generate CARs. At last, the qualified CAR-T cells were infused into relapse subjects to kill the leukemia cells. CAR chimeric antigen receptor, BMC blood mononuclear cells.

The method for obtaining anti-CD19 CAR-T cells is previously described [7]. Briefly, blood mononuclear cells were obtained from transplant donors by leukapheresis, T cells were purified and transfected with lentivirus containing sequence expressing chimeric antigen receptors (CARs) with the 4-1BB or CD28 intracellular domain as co-stimulation signal and expanded in vitro. Quality control was based on the Chinese Pharmacopoeia (2015 Version), which includes viability >70% (Fig. 1).

### CD19-targeting CAR-T cell therapy

Three preinfusion immune suppressive regimens were: (1) regimen 1: fludarabine, 30 mg/mE + 2/day for 2–4 days and cyclophosphamide, 200 mg/mE + 2/day for 2 days; (2) regimen 2: fludarabine, 30 mg/mE + 2/day for 3 days, cyclophosphamide, 350 mg/mE + 2/day for 2 days and cytarabine, and 100 mg/mE+2/day for 4 days; and (3) regimen 3: cyclophosphamide, 500 mg/mE + 2/day for 3 days. Overall, 34 subjects received regimen 1. Regimen 2 was given to five younger subjects with many leukemia cells. Regimen 3 was given to four older subjects with few leukemia cells. Expansion and persistence of CAR-T cells were analyzed by flow cytometry or by CAR-T cell DNA copy number [7]. Median dose of infused CAR-T cells was 1.76 × 10E + 6/kg (range, 0.4–12 × 10E + 6/kg).

### Response assessment

Complete remission was defined by histology (<5% bone marrow blasts) and a negative MRD test (<0.01% bone marrow blasts) assessed by multiparameter flow cytometry to detect the leukemia-associated profiles [8, 10]. It also included normal maturation of all cell components in the bone marrow, no extramedullary leukemia (e.g. central nervous system, testes or soft tissue), blood neutrophil concentration ≥1 × 10E + 9/L, blood platelet concentration ≥100 × 10E + 9/L and RBC and platelet transfusion independence.

Relapse included histologic and a positive MRD test. Histologic relapse was defined as blasts ≥5% in blood or bone marrow and/or in an extramedullary site after achieving a complete histologic remission post transplant. Molecular relapse was defined as a positive MRD test (>0.01% and <5%) without evidence of histologic relapse post transplant [11].

### Adverse events

Grading of acute G*v*HD was based on published criteria [12, 13], as was grading of cytokine release syndrome (CRS) [14]. Immune effector cell-associated neurotoxicity syndrome (ICANS) was graded using the Common Terminology Criteria for Adverse Events [15]. CRS, ICANS, and acute G*v*HD were managed as previously described [7, 14, 15].

### Statistics

The time of CAR-T cell transfusion was used as the origin in all the time-to-event analyses. Analysis of CIR used relapse as the event. For analysis of EFS, no response, relapse or death, whichever occurred first, was regarded as the event. In survival analyses, death was the event. Subjects without an event were censored at the date they were last known to be alive. Two subjects receiving a second allotransplant were censored at the time of second transplant. The primary study endpoints were safety and efficacy. Secondary endpoints were covariates associated with safety and efficacy. Data were analyzed as of September 30, 2019 with a median follow-up of survivors of 17 months (range, 6–47 months).

The chi-square statistic or Fisher exact test was used for comparisons between categorical variables, and the Mann–Whitney *U* test was used for continuous variables. The Kaplan–Meier method was used to calculate the probability of EFS and survival. *P* values were two-sided, and *P* < 0.05 was considered significant. SPSS statistical software for Windows, version 24.0 (SPSS, Chicago, IL, USA) was used for statistical analyses.

## Results

### Subjects

Forty-three subjects were enrolled and analyzed (Table 1). Twenty-nine were male. Median age was 24 years (range, 4–60 years). Pretransplant conditioning regimens are displayed in Table 1. Donors were HLA-identical siblings (*N* = 17) or HLA-haplotype-matched relatives (*N* = 26). Bone marrow blasts at relapse were 0.01–5% in 13 subjects, 5–50% in 20 and >50% in 10 subjects. The median interval from relapse to CAR-T cell infusion was 42 days (range, 35–59 days). Postrelapse therapies included stopping immune suppression (*N* = 12), DLI (*N* = 7), chemotherapy (*N* = 19), and DLI and chemotherapy (*N* = 5; Supplementery Table 1).

**Table 1:** Patient covariates (*N* = 43).

Male	29
Age (Median; range)	24 (4–60)
Donor	
HLA-identical sibling	17
HLA-haplotype-matched	26
Graft	
Blood	17
Blood and bone marrow	26
*BCR/ABL1* positive	
Yes	5
No	38
Cytogenetics	
Abnormal	14
Normal	29
Mutation	
Yes	23
No	20
Transplant conditioning regimen	
BU/CY	17
BU/CY/Ara-C/CCNU	26
Posttransplant immune suppression	
CSA/MMF/MTX	17
Tacrolimus/MMF/ATG/MTX	26
Interval from transplant to relapse (mo; median; range)	8 (1–25)
Interval from relapse to CAR-T (d; median; range)	42 (35–59)
Therapy for relapse	
Stop immune suppression	12
DLI	7
Chemotherapy	19
Chemotherapy/DLI	5
Bone marrow blasts pre infusion	
0.01-(MRD-positive)	13
5–50%	20
>50%	10
Co-stimulatory molecular	
CD28	18
4–1BB	25
Preinfusion therapy	
Regimen 1	34
Regimen 2	5
Regimen 3	4
CAR-T cell dose (×10E + 6/kg; median; range)	1.76 (0.4–12)
<1	4
1~2	26
>2	13

*HLA* human leukocyte antigen, *BU* busulfan, *CY* cyclophosphamide, *Ara-C* cytarabine, *CCNU* lomustine, *CSA* cyclosporine, *MMF* mycophenolate mofetil, *MTX* methotrexate, *ATG* anti-thymocyte globulin, *MRD* measurable residual disease, *DLI* donor lymphocyte infusion.

Overall, 18 subjects received CD19–28z CAR-T cells and 25, CD19-BBz CAR-T cells. Pre infusion, 34 subjects (79%) received regimen 1, 5 regimen 2, and 4, regimen 3. Median numbers of infused CAR-T cells were 1.76 × 10E + 6/kg (range, 0.4–12 × 10E + 6/kg; Table 1).

### Safety

All subjects had a decreased concentration of hemoglobin, WBCs, neutrophils, lymphocytes, and platelets. One subject had an increased activated partial thromboplastin time. Two subjects had an increased alkaline phosphatase, alanine aminotransferase, aspartate aminotransferase, and lactic dehydrogenase levels.

CRS of any grade developed in 38 subjects (88%; 95% confidence interval (CI). 78, 98%) and was ≥grade-3 in 7 subjects. Six of seven subjects with ≥grade-3 CRS had bone marrow blasts >5% including four with bone marrow blasts >50% and two, 20–50%. There was no significant correlation between risk of severe CRS or incidence or severity of ICANS and percentage bone marrow blasts. Overall, 16 of 18 subjects receiving CD19–28z CAR-T cells developed CRS, severe in 5. 22 of 25 subjects receiving CD19-BBz CAR-T cells developed CRS, severe in 4. 3 of 18 subjects receiving CD19-28z CAR-T cells developed ICANS compared with 6 of 25 receiving CD19-BBz CAR-T cells (*P* = 0.79). Two subjects receiving CAR-T cells developed ≤grade-2 acute G*v*HD. Nine subjects (21% [8, 34%]) developed grade-1/-2 ICANS with none ≥grade-3.

### Efficacy

41 subjects survived ≥21 days and were evaluable for response. Two died in <21 days from CRS and multiorgan failure on days 14 and 21 and were included in the *intent-to-treat* analysis. 34 subjects (79% [66, 92%]) achieved a complete histological remission including 12 of 13 with 0.01–5% bone marrow blasts and a positive MRD test, 14 of 20 with 5–50% bone marrow blasts and 8 of 10 with ≥50% bone marrow blasts. Although there are no significant differences in rates between the cohorts, the comparison is not adjusted for other covariates such as the preinfusion regimen or type of CAR-T cells. 14 of 18 subjects receiving CD19–28z CAR-T cells achieved a complete histological remission compared with 20 of 25 receiving CD19-BBz CAR-T cells. Because therapy assignment was not random and not adjusted for other covariates, we did not compare these rates statistically.

The 34 subjects achieving a complete histological remission received different CAR-T doses, including two of four receiving <1 × 10E + 6, 23 of 26 receiving 1–2 × 10E + 6, and 9 of 13 receiving >2 × 10E + 6. Because therapy assignment was not random, we did not compare these rates statistically. These 34 received different preinfusion regimens, including 26 receiving regimen 1, five of five receiving regimen 2, and three of four receiving regimen 3.

**Fig. 2:**
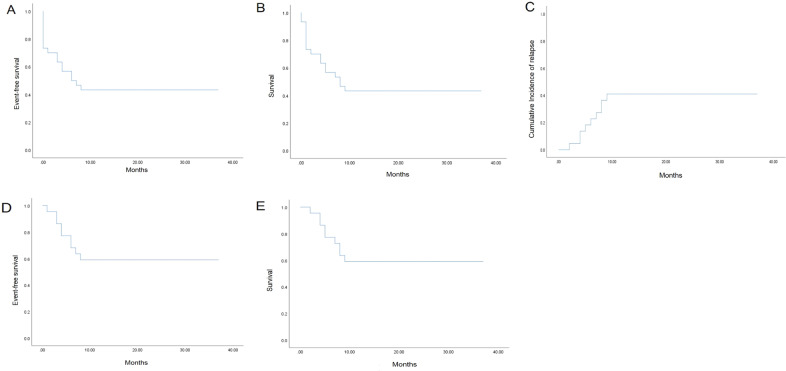
Survival after CD19 donor-derived CAR-T cell treatment of relapsed B-ALL after an allotransplant. **a** 1-year EFS; **b** 1-year survival; **c** 1-year CIR in subjects with a complete histological remission without a second transplant; **d**, **e** 1-year EFS and survival in subjects with a complete histological remission without a second transplant. EFS event-free survival, CIR cumulative incidence of relapse.

1-year probabilities of EFS and survival were 43% (25, 62%; Fig. 2a, b). 1-year probability of CIR was 41% (25, 62%) in subjects achieving a complete histological remission not receiving a second transplant (Fig. 2c). In the 32 subjects achieving a complete histological remission not receiving a second transplant 1-year probabilities of EFS and survival were 59% (37, 81%; Fig. 2d, e). Two of nine subjects not achieving a complete histological remission lost CD19 expression. Three other subjects who relapsed lost CD19 expression.

### CAR-T cell dynamics

CAR-T cell numbers peaked on day 9 (range, day 3–61) after CAR-T cell infusion in the 36 subjects with complete data. Median interval of detectable blood CAR-T cells post infusion was 89 days (range, 10–1230 days). Postinfusion CAR-T cells peaked on day 10 (range, days 3–61) and 9 (range, days 7–21) in subjects with and without a complete histological remission, respectively. Corresponding median postinfusion intervals of detectable blood CAR-T cells were 118 days (range, 10–1230 days) and 21 days (range, 14–28 days). Median peak concentration was 4.85 × 10E + 5/L (range, 0.14–1.18 × 10 + 5/L). Median peak percentage of all cells was 23% (range, 2–65%).

## Discussion

Our data indicate that donor-derived anti-CD19 CAR-T cells are a safe and effective therapy for B-ALL recurrence after allotransplantation. Adverse effects of CAR-T cell therapy are mainly CRS and ICANS, severity of which reportedly correlates with cancer volume, preinfusion regimen and CAR-T cell dose in some but not all studies [16, 17]. We found no such correlations but we had relatively few subjects and many confounding covariates. Others have reported similar data [18, 19]. Although the incidence of CRS in our study is highly compared with studies of autologous CAR-T cells, most cases were low-grade and controllable. Absent a randomized comparison any conclusion is tentative. Nine subjects developed grade-1/-2 ICANS with none ≥grade-3. This rate is like that reported after autologous CAR-T cell therapy [20].

Donor-derived CAR-T cell treatment can potentially cause acute G*v*HD. In a mouse model, donor-derived CAR-T cells had little G*v*HD but a potent graft-*versus*-leukemia (G*v*L) effect [21]. A systematic review reported a low risk of acute G*v*HD after allogeneic CAR-T cells [22]. Two subjects in our study developed mild acute G*v*HD. These data imply an ability to separate G*v*HD from G*v*L, albeit in an artificial setting.

There are a few reports of using allogeneic CAR-T cells to treat B-cell cancers after an allogeneic hematopoietic stem cell transplant (Table 2) [18, 23–29]. No study had >30 subjects. Moreover, most subjects in these reports received a second transplant making critical analyses of safety and efficacy of the CAR-T cell infusion impossible. We censored data from subjects who received a second transplant, making it possible to ascertain the safety and efficacy of CAR-T cell therapy alone.

**Table 2:** Reports of donor CAR-T cells given for relapse of B-cell cancers after an allotransplant.

Subjects (*N*)	Age^a^	B-ALL		T-ALL (CD19^+^)	B-CLL	Lymphoma				Ref.
		Ph^1+^ or *BCRABL1*^*+*^	Ph^1−^ or *BCRABL*1^−^			MCL	DLBCL	Follicular	Nodular HL	
8	32 (9–59)	2	2		4					[23]
10	52 (44–66)				4	4	2			[24]
16	50 (23–74)	4	12							[25]
9	39 (15–64)	5	4							[26]
20	46 (20–68)	1	4		5	5	5			[27]
26	40 (21–61)	17				1	4	3	1	[28]
30	14 (5–60)	2	27	1						[29]
6	27 (8–44)	1	5							[18]

*Ph*^*1*^ Philadelphia1-chromosome, *BCRABL1* qRT-PCR result, *Ref* reference.

^a^Years (y); median; range.

Other potential therapies of relapse after an allotransplant include stopping immune suppression, receiving DLI and/or receiving a second transplant from the same or a different donor. We summarize the data using these strategies in Table 3. It was difficult to compare our outcomes with these other strategies. There are only four studies of DLI, three of which had ≤10 subjects. There were only five studies of a second transplant only two of which had many subjects and none indicated consecutive subjects. Studies of stopping posttransplant immune suppression were typically confounded by combination with other therapies, often given concurrently. These limited data and confounding factors make it impossible to critical compare our data with those from other studies. A randomized trial is needed to resolve this question but is highly unlikely.

**Table 3:** Therapies of relapse of ALL post allotransplant.

	*N*	ALL		Age^a^	CR	Stop immune suppression^b^	CIR	EFS	Survival	aG*v*HD ≥ grade 2	Ref.
		B	T								
This study	43	43	**─**	24 (4–60)	79%	**─**	57%	43%	43%	0	
DLI	10	**─**	**─**	11 (< 1–25)	**─**	N/A	N/A	N/A	**─**	N/A	[30]
DLI	30	**─**	**─**	21 (10–52)	25%	N/A	N/A	N/A	5%	N/A	[31]
DLI	8	**─**	**─**	24 (18–39)	**─**	5 (2–14)	N/A	**─**	**─**	5	[32]
DLI	10	**─**	**─**	33 (18–40)	70%	N/A	N/A	N/A	**─**	6	[33]
Second transplant	245	186	59	35 (18–74)	N/A	N/A	56%	20%	30%	127	[34]
Second transplant	11	**─**	**─**	41 (18–65)	N/A	Tapered at 3 mo	N/A	**─**	**─**	N/A	[35]
Second transplant	214	**─**	**─**	8 (1–18)	78%	N/A	44%	34%	43%	53	[36]
Second transplant	27	22	5	37 (7–60)	N/A	N/A	56%	30%	41%	15	[37]
Second transplant	31	**─**	**─**	26 (7–49)	75%	N/A	N/A	N/A	23%	13	[38]

Estimates for studies with <30 subjects are unreliable with wide 95% confidence intervals so complete histological remission rates, CIR, EFS, and survival are not indicated.

*CR* complete histological remission, *DLI* donor lymphocyte infusion, *EFS* event-free survival, *GvHD* graft-versus-host disease, *Ref* reference, *N/A* not available.

^a^Year; median range.

^b^Month; median; range.

Our study has important limitations. We had relatively few subjects who with different diagnoses received diverse postrelapse interventions before CAR-T cell therapy. They also received different preinfusion regimens, different CAR-T cell constructs and different doses which preclude us from making definitive conclusions regarding subject-, disease- and therapy-related covariates correlated with outcomes. Consequently, we refrained from comparing outcomes of covariates such as preinfusion regimen and type and dose of CAR-T cells. Results of such comparisons are likely to be confounded by known and latent (unknown) covariates and small sample sizes, are unreliable and are not statically justifiable. Also, because our subjects are not consecutive cases of everyone relapsing at the study centers there are important selection biases. Our study was retrospective, and participating center admission records were not independently audited.

It should also be noted that, CAR-T cell manufacturing in different medical centers may cause heterogeneity, but every procedure complies with a rigorous quality control, which is consistent. The outcomes of our study with the largest number of relapsed ALL subjects are satisfactory, donor-derived CAR-T is a good choice for those who are not eligible for receiving a second transplant.

In summary, we show therapy of recurrent B-ALL after an allotransplant with donor-derived anti-CD19 CAR-T cells is safe and effective. Outcomes seem comparable to those achieved with alternative therapies. However, the relative safety and efficacy of these alternatives can be accurately determined only in a randomized trial.

## Supplementary information

Supplemental Table 1
