# Supplementary material for: Donor-derived CD19 CAR-T cell therapy of relapse of CD19-positive B-ALL post allotransplant
Source: Leukemia. 2020 Oct 19;35(6):1563–70. doi: 10.1038/s41375-020-01056-6 (PMC8179843; doi:10.1038/s41375-020-01056-6)
Supplement: Supplementary file 1 — Supplemental Table 1 [file 41375_2020_1056_MOESM1_ESM.docx]

**Supplemental Table 1** Therapies for relapse pre-infusion

| Therapy | N |  |
| --- | --- | --- |
| Stop immune suppression | 5 | Cyclosporine |
| Stop immune suppression | 7 | Tacrolimus |
| DLI | 7 | 1×10E+8 |
| CVTLP | 19 | N/A |
| CVTLP//DLI | 5 | 10E+8 |

DLI, Donor lymphocyte infusion; CVTLP, cyclophosphamide, vindesine, pirarubicin, prednisone and peg-asparaginase.
